# Differential requirement of cyclin-dependent kinase 2 for oligodendrocyte progenitor cell proliferation and differentiation

**DOI:** 10.1186/1747-1028-7-14

**Published:** 2012-05-14

**Authors:** Céline Caillava, Anne Baron-Van Evercooren

**Affiliations:** 1 Université Pierre et Marie Curie-Paris 6, Centre de Recherche de l’Institut du Cerveau et de la Moelle Epinière, UMR-S975, Paris, France; 2 Inserm, U975, Paris, France; 3 Cnrs, UMR 7225, Paris, France; 4 AP-HP Hôpital Pitié-Salpêtrière, Fédération de Neurologie, Paris, France

**Keywords:** Cdk2, Proliferation, Remyelination, Oligodendrocyte progenitor, Adult neural stem cell

## Abstract

Cyclin-dependent kinases (Cdks) and their cyclin regulatory subunits control cell growth and division. Cdk2-cyclin E complexes, phosphorylating the retinoblastoma protein, drive cells through the G1/S transition into the S phase of the cell cycle. Despite its fundamental role, Cdk2 was found to be indispensable only in specific cell types due to molecular redundancies in its function. Converging studies highlight involvement of Cdk2 and associated cell cycle regulatory proteins in oligodendrocyte progenitor cell proliferation and differentiation. Giving the contribution of this immature cell type to brain plasticity and repair in the adult, this review will explore the requirement of Cdk2 for oligodendrogenesis, oligodendrocyte progenitor cells proliferation and differentiation during physiological and pathological conditions.

## Review

During development, the majority of oligodendrocyte progenitor cells (OPCs) undergo a limited period of proliferation, before cell cycle exit and terminal differentiation into postmitotic myelinating oligodendrocytes [1]. However, a population of slowly dividing OPCs persists throughout the adult CNS [2-4] and there is growing interest concerning the signals influencing proliferation and differentiation of these progenitors [5]. Adult OPCs share common features with perinatal OPCs such as the expression of NG2 and PDGFRα and the ability to differentiate into oligodendrocytes but also astrocytes and neurons [4,6-11]. However, their cell cycle length and rate of differentiation are slower comparing with perinatal OPCs. They exhibit precise proliferative capacity defined as intrinsic timer concept with each cell dividing invariably 8 times in culture before exiting the cell cycle and differentiating [1,12]. Moreover, despite their low oligodendrogenic potential under physiological conditions, adult OPCs can be reactivated in terms of proliferation, migration and differentiation leading to remyelination after CNS white matter insults [4,13-15]. Therefore they are considered as major targets for therapeutical strategies. OPCs can also be generated from the subventricular zone (SVZ) in the adult forebrain both under physiological conditions [16,17] and after demyelination in rodents and human brain [18-21]. After demyelination of the adult brain, SVZ-derived OPCs are recruited to the lesions and contribute to periventricular remyelination [22,23]. All these specificities make the study of the cell cycle regulation in this cell type of great interest.

**Figure 1:**
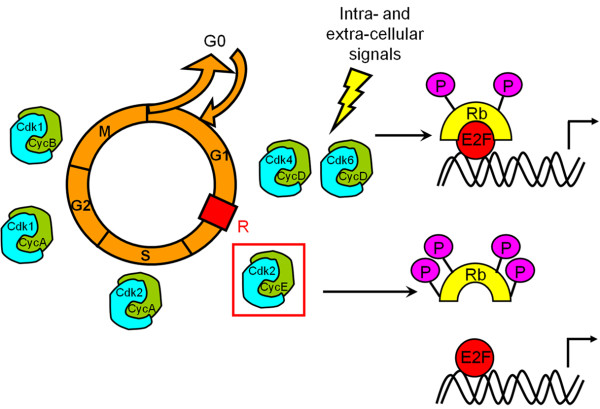
**Control of cell cycle progression.** Mitogenic signalling results in cyclin D synthesis, formation of active Cdk4/6-cyclin D complexes and initial phosphorylation of Rb. Partially phosphorylated Rb still binds to transcription factor E2F but some genes can be transcribed, such as cyclin E which binds to and activates Cdk2. It is generally accepted that Cdk2-dependent phosphorylation of Rb results in its complete inactivation, which allows induction of the E2F-responsive genes that are needed to drive cells through the G1/S transition. R represents the restriction point that separates the mitogen-dependent early G1 phase from the mitogen-independent late G1 phase. Cdk2 associated to cyclin A controls progression through S phase. Cdk1-cyclin A complex is responsible for G2 phase progression. Cdk1 associated to cyclin B is essential for both G2/M transition and mitosis progression.

Cell proliferation is a key phenomenon during development and repair. It is regulated by complex extrinsic and intrinsic mechanisms involving cyclin-dependent kinases (Cdks), a family of serine/threonine kinases that represents the core of the cell cycle machinery. Successive waves of Cdk activity control initiation and progression through the eukaryotic cell cycle in response to both intra-cellular and extra-cellular signals (Figure 1). During early G1 phase, the retinoblastoma protein Rb acts as a negative regulator of the cell cycle through its ability to sequester the transcription factor E2F. Later, its phosphorylation initiated by Cdk4/6 and completed by Cdk2 - activated upon specific binding of the D and E cyclins, respectively [24] - irreversibly inactivates Rb. E2F transcription factor are going to be released and translocate to the nucleus to promote transcription of genes essential for S-phase entry including E cyclin. G1/S transition no longer depends on mitogen factors: activity of Cdk2-cyclin E complexes enables to reach the restriction point. Giving their key role in the progression of the cell cycle, Cdk activity is tightly regulated at different levels to fit to both intra-cellular and extra-cellular signals. This regulation process includes interactions with activating subunits (the cyclins), Cdk inhibitors or Cdkis (INK4 and Cip/Kip family members), phosphorylation-dephosphorylation, folding and subcellular localization. INK4 proteins (p16^INK4a^, p15^INK4b^, p18^INK4c^ and p19^INK4d^) specifically interact with Cdk4 and Cdk6 and prevent their association with D cyclin. Cip/Kip proteins (p21^Cip1^, p27^Kip1^ and p57^Kip2^) have a broader spectrum of activity. Associated to Cdk2, they block its kinase activity but, in return, Cdk2 associated kinase activity regulates the proteolysis of both its activator E cyclin and its inhibitor p27^Kip1^[25]. First observations demonstrated the essential requirement of p21 and p27 in stoechiometric proportions for the formation of Cdk4/6-cyclin D complexes [25] but were not confirmed [26,27]. After formation of Cdk4/6-cyclin D complexes in the cytoplasm and import to the nucleus, these complexes are going to sequester p27 from resident Cdk2/cyclin E/p27 complexes allowing Cdk2 activation [28]. Expression of some Cdk inhibitors can be directly or indirectly regulated by the tumour suppressor p53. Because of the specificity of adult OPCs cell cycle regulation and their reactivation potential, several groups have investigated the links between cell cycle regulation and oligodendrocyte differentiation [1,12,29-36]. In this review, we discuss the specific role of Cdk2 as an important component of the mechanism that couples the regulation of OPC proliferation and differentiation.

### Differential requirement for Cdk2 in adult germinative regions

#### Cdk2 is critical for proliferation and self-renewal of neural progenitor cells in the adult subventricular zone

Adult oligodendrocytes can arise from either white matter OPC or neural progenitor cells from the subventricular zone (SVZ). Therefore a study investigated the function of Cdk2 in the adult subventricular zone and more precisely in chondroitin sulfate proteoglycan NG2-expressing progenitors taking advantage of a viable Cdk2−/− mouse mutant [37,38]. NG2^+^ cells from the SVZ display cellular properties of transient-amplifying (type C) cells and are able to generate oligodendrocytes in the adult [6,22]. Proliferation of NG2^+^ cells was demonstrated to be selectively impaired in the adult SVZ of mice lacking Cdk2 (Table 1). Importantly, loss of Cdk2 did not affect proliferation of slowly dividing GFAP^+^-nestin^+^ stem cells or PSA-NCAM^+^ neuroblasts [30,39] indicating that intrinsic cell cycle regulators play distinct roles in different cell populations of the adult SVZ. Analysis in cultures obtained from adult SVZ NG2^+^ cells is consistent with the *in vivo* findings, as a significant decrease in the formation of neurospheres was also observed in Cdk2−/− cells [39]. Moreover, it appears that Cdk2 might play a specific role in adult neural progenitors because loss of this kinase did not affect proliferation of embryonic fibroblasts or human colon cancer cell lines in culture [37,38,40].

**Table 1:** Differential influence of Cdk2 loss on OPC

	**Physiological condition**		**Pathological condition**	
	**SVZ**	**CC**	**SVZ**	**CC**
**Proliferation**	**↓**	**NO**	**↓**	**↓**
**Differentiation**	**↑**	**NO**	**↑**	**↑**
**Myelination/ remyelination**	**NC**	**NO**	**NC**	**↑**

The pathological condition concerns corpus callosum LPC-induced demyelination. *SVZ* subventricular zone; *CC* corpus callosum; *NO* not observed; *NC* not concerned

The decrease in NG2^+^ cell proliferation observed in the adult SVZ may result from a shift in balance between cell proliferation and differentiation and/or cell death. Caspase-3^+^ apoptotic cells were quantified and no differences were observed in cell death in the absence of Cdk2 at P8 or P90. These results were confirmed by quantification of TUNEL^+^ cells [39]. However, cellular and molecular analyses *in vivo* and *in vitro* demonstrate that the loss of Cdk2 promotes NG2^+^ cell lineage commitment and differentiation in oligodendrocytes of adult SVZ cells [39] (Table 1). Regarding these results, Cdk2 appears to contribute not only to cell cycle regulation but also to the decision to differentiate. At variance with findings in the adult SVZ, *in vivo* and *in vitro* analysis demonstrated that both NG2^+^ cell proliferation and self-renewal capacities were not affected by the loss of the Cdk2 gene up to postnatal day 15 implying during perinatal period compensatory activity of other Cdks which is a well known phenomenon [41]. Cdk1 could play this role as in the absence of interphase Cdks (Cdk4, Cdk6 and Cdk2), it can execute all the events that are required to drive mammalian cell division [42]. More precisely, in p27−/−; Cdk2−/− double KO mice, Cdk1 compensates the loss of Cdk2 function, binding to cyclin E and regulating G1/S transition [37,43]. However, probably due to the importance of the genetic locus for Cdk function [44] in specific cell types, Cdk1 is unable to compensate for the loss of Cdk2 in germinal cells as Cdk2−/− mice are sterile [37,38]. In SVZ protein extracts from Cdk2−/− P8 and P90 mice, Cdk1 expression was evaluated and difference with wild-type mice could not be found. Actually, compensatory mechanisms in perinatal Cdk2−/− SVZ cells, which persist until postnatal day 15, involve increased Cdk4 expression that results in retinoblastoma protein inactivation [39]. A subsequent decline in Cdk4 activity to wild-type levels in postnatal day 28 Cdk2−/− cells coincides with lower NG2^+^ proliferation and self-renewal capacity similar to adult levels. Cdk4 compensation was confirmed by silencing experiments in perinatal Cdk2−/− SVZ cells that abolishes Cdk4 up-regulation and reduces cell proliferation and self-renewal to adult levels. Conversely, Cdk4 overexpression in adult SVZ cells restores proliferative capacity to wild-type levels [39]. Thus, although Cdk2 is functionally redundant in perinatal SVZ, it is important for adult progenitor cell proliferation and self-renewal, through age-dependent regulation of Cdk4.

#### Cdk2 is dispensable for adult hippocampal neurogenesis

The subventricular zone does not constitute the only persistant germinative zone in the adult as granule neurons undergo continuous renewal throughout life in the dentate gyrus (DG) of the hippocampus. Thus, the requirement of Cdk2 has also been investigated in this region using Cdk2 deficient mice [45]. Surprisingly, the quantification of cell cycle markers first revealed that the lack of Cdk2 activity does not influence spontaneous or seizure-induced proliferation of neural progenitor cells in the adult DG. Using bromodeoxyuridine incorporation assays, it was shown that the number of mature newborn granule neurons generated *de novo* was similar in both wild-type and Cdk2-deficient adult mice. Moreover, the apparent lack of cell output reduction in Cdk2−/− mice DG did not result from a reduction in apoptosis of newborn granule cells as analyzed by TUNEL assays [45]. So, contrary to its role in NPC proliferation in the adult SVZ, Cdk2 seems to be dispensable for NPC proliferation, differentiation and survival of adult-born DG granule neurons *in vivo*. This discrepancy could be explained by compensatory mechanisms, which may be region-specific and may reflect the low proliferative potential of hippocampal progenitors, as compared to their SVZ counterpart. Alternatively, these differences may have resulted from a non essential role played by Cdk2 in neurogenic regions of the brain, such as the adult hippocampus, as compared to the SVZ, which is directly involved in oligodendrogenesis, a process that more closely depends on the control of OPC proliferation by Cdk2.

### Influence of Cdk2 and related cell cycle proteins on OPC

Martin C. Raff was one of the first to identify integrated sequential signals that control oligodendrocyte differentiation. He postulated that a cell-intrinsic timer regulates the onset of oligodendroglial differentiation based on a model system in which the normal timing of oligodendrocyte development can be reconstituted in OPC cultures of dissociated embryonic and perinatal optic nerve, as long as OPCs are stimulated to divide by PDGF [35,46]. Indeed, in these conditions, OPCs divided up to eight times before they stopped and differentiated [1,12]. The question of how the timer works is still unanswered but several candidate factors have been identified to be part of the molecular structure of this cell intrinsic timer and all the studies converge to proteins involved in G1/S transition and especially Cdk2 [5].

Inhibitors that belong to INK family can play a role in OPCs proliferation. Indeed, accumulation of p18^INK4c^ in OPCs was demonstrated to precede proliferation arrest and its overexpression can prevent OPCs proliferation induced by mitogens [47]. Actually, high levels of INK4 proteins inhibit the activity of Cdk4/6-cyclin D complexes which can no more sequestrate Cip/Kip inhibitors that are going to inhibit Cdk2-cyclin E complexes and then induce OPCs cell cycle arrest.

Depending on the levels and modality of activation, the tumor suppressor p53 can act as transcriptional activator or repressor of many genes, causing apoptosis, cell cycle arrest in G1 or G2/M, or differentiation depending on the cell type [48]. In proliferating OPCs, p53 proteins are low and the protein is likely not functional, because of its cytoplasmic localization [49]. Upon removal of mitogens, however, p53 is transiently translocated to the nucleus and maybe involved in both growth arrest and the onset of OPC differentiation by stimulating p21 expression and consequently inhibition of Cdk2-cyclin E complexes [49].

**Figure 2:**
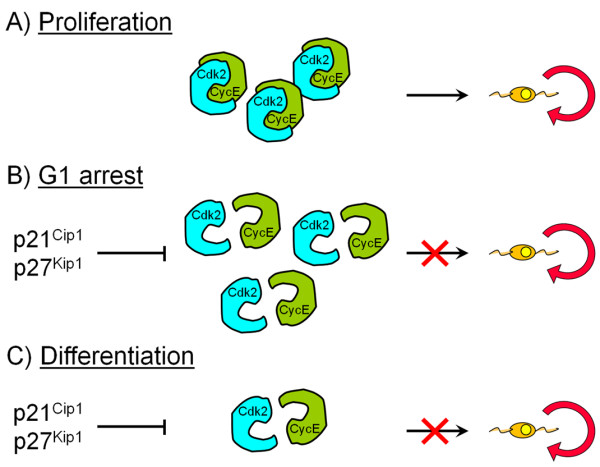
**Summary scheme describing Cdk2-cyclin E interactions involved in cell cycle withdrawal and arrest in OPC.** Compared to proliferating OPCs **(A)**, Cdk2 and cyclin E protein expression is not modified during reversible cell cycle arrest in G1 **(B)** but Cdk2-cyclin E complex formation is impaired which causes a selective decrease in Cdk2 activity. However, in differentiated cells that had permanently withdrawn from the cycle **(C)**, protein levels, Cdk2-cyclin E complex formation and kinase activity are decreased compared to proliferating OPCs (A).

Previous studies *in vitro* and *in vivo* indicated that p27^Kip1^ and p21^Cip1^ that negatively regulate the activity of the Cdk 4/6-cyclin D and Cdk2-cyclin E complexes [50], are important regulators of OPC proliferation during development [31,32,34,51-54]. The levels of these Cdkis increase in OPCs either during permanent cell cycle withdrawal and differentiation or during reversible cell cycle arrest in G1 caused by neuronal signals [31,32,34,51-54] (Figure 2). Regulation of G1 phase progression was consequently supposed to be crucial for OPC proliferation. The increase in p27^Kip1^ protein levels observed during OPC differentiation results in an enhancement of its binding to the Cdk2-cyclin E complex and inhibition of Cdk2 activity [54,55]. Ectopic expression of p27^Kip1^ in OPCs causes a significant reduction in Cdk2-cyclin E activity and cell cycle arrest [36,56]. Purified rat OPCs were used to investigate the molecular mechanisms that regulate permanent cell cycle withdrawal and reversible arrest in the oligodendrocyte lineage [33]. Higher protein levels of cyclin E and D and Cdks 2, 4 and 6 and higher kinase activities of both Cdk4/6-cyclin D and Cdk2-cyclin E were found in proliferating OPCs compared to cells that had permanently withdrawn from the cycle. This was associated with a decrease in the formation of the Cdk2-cyclin E and Cdk4/6-cyclin D complexes in differentiated oligodendrocytes [33]. Reversible cell cycle arrest in G1 induced by glutamatergic and β-adrenergic receptor activation or cell depolarization, however, did not modify cyclin E and Cdk2 protein expression compared with proliferating OPCs. Instead, these agents caused a selective decrease in Cdk2 activity and an impairment of Cdk2-cyclin E complex formation. Although cyclin D protein levels were higher than in proliferating cells, cyclin D-associated kinase activity was not modified in G1-arrested OPCs [33]. These results are consistent with analysis in corpus callosum *in vivo* that showed that Cdk2-cyclin E activity increased between postnatal days 3 and 15 and decreased between postnatal days 15 and 30, during and after terminal division and differentiation of immature oligodendrocytes [33]. These results indicate that the Cdk2-cyclin E complex is a major regulator of OPC cycle progression and that the Cdks involved in reversible cell cycle arrest are distinct from those implicated in permanent cell cycle withdrawal. To establish a causal link between Cdk2-cyclin E activity and OPC proliferation, Cdk2 activity was selectively modulated *in vitro* by transfection of cultured OPCs [29]. Dominant-negative (Dn)-Cdk2 overexpression inhibited mitogen-induced OPC proliferation, whereas wild-type (WT)-Cdk2 prevented cell cycle arrest caused by anti-mitotic signals. Therefore, in OPCs, cell cycle pathways activated either in a pro- or in anti-mitotic environment, converge on the common molecular target Cdk2. Dn-Cdk2- or WT-Cdk2-mediated regulation of G1/S transition, *per se*, did not influence initiation of OPC differentiation.

*In vitro* studies demonstrated lineage continuity between perinatal and adult OPCs [8,11]. However, the molecular basis of their differences in cell proliferation and cell cycle length are still unknown [57]. To study the function of Cdk2-cyclin E in OPCs during development *in vivo*, Cdk2 and cyclin E expression were first analyzed in cells acutely isolated from transgenic mice expressing GFP under the control of CNP gene promoter. In these transgenic mice, GFP is selectively expressed in the oligodendroglial lineage throughout embryonic and postnatal development [58,59]. Both Cdk2-cyclin E protein levels and activity were found decreased in GFP^+^ oligodendrocyte lineage cells between postnatal days 4 and 30. Immunostaining of NG2^+^/GFP^+^ OPCs in brain tissue sections showed a 90% decrease in overall cell proliferation and Cdk2 expression between perinatal and adult cells [29,30]. However, Cdk2 expression within the proliferating OPC population was maintained throughout development. Intrinsic differences in Cdk2-cyclin E expression and activity may underlie the slowly proliferative state that characterizes the so-called quiescent adult OPCs *in vivo*. Cdk2-deficient mice were also analyzed to elucidate the functional involvement of Cdk2 in OPC proliferation and oligodendrogenesis *in vivo*. Interestingly, loss of Cdk2 had no effect on OPC proliferation and generation of oligodendrocytes during early postnatal (P8) development and in the adult (P90) corpus callosum [30]. Moreover, differences in density and distribution of cells representing different stages of oligodendrocyte development were not observed in WT versus Cdk2−/− adult mice (P90). These results suggest that, even if Cdk2 controls OPC proliferation, it is not required for normal development of mature oligodendrocytes in the postnatal and adult CNS (Table 1). This discrepancy could be explained by compensatory mechanisms as described in the SVZ, only effective during development and early postnatal, whereas requirement for Cdk2 is less important in the adult due to the low proliferative potential of adult OPC.

### Cdk2 and cell cycle regulation in white matter disorders

#### Differential influence of Cdk2 on CNS myelination and remyelination

The data described previously indicate that Cdk2 loss does affect neither the number of white matter oligodendrocytes and OPCs, nor their proliferation rate. However, since oligodendrocytes are responsible for myelin sheath synthesis with each cell myelinating up to 40 independent axonal segments [60], loss of Cdk2 could impact CNS myelin synthesis. To explore this possibility, electron microscopy was used but did not reveal any changes in axonal preservation, in the number of myelinated axons, or differences in myelin structure, after Cdk2 loss in the postnatal (P15) or adult (P90) corpus callosum, since myelin compaction and g ratio did not significantly differ in Cdk2−/− and WT mice [30]. In addition, Western blot quantification of MBP, one of the most abundant CNS myelin protein [61], showed no difference in its levels between the two genotypes. Taken together, these data suggest that myelination during early postnatal development is either Cdk2-independent or is efficiently compensated by other Cdks (Table 1).

Although some OPCs are still produced in the adult from SVZ cells [10,22,62], decreased proliferation of these cells under physiological conditions in the absence of Cdk2 [39] seems sufficient to maintain a normal density of oligodendroglial cells in the corpus callosum. However, when challenged with demyelination induced by LPC injection, a process known to enhance proliferation of resident adult OPCs and SVZ precursors [18,21], cell proliferation was found to be reduced not only in the SVZ, but also in the lesion of Cdk2−/− mice [30] (Table 1). These alterations were not an indirect consequence of axonal loss, as no obvious alteration in axons and/or in axonal density, were observed by electron microscopy. Since decreased proliferation specifically affected cells giving rise to oligodendrocytes, including OPCs and type C cells of the SVZ, rather than microglia and astrocytes, the consequences of altered proliferation on OPC differentiation into oligodendrocytes were investigated. Reduced proliferation was shown to be correlated with enhanced cell cycle exit in adult OPCs of Cdk2 −/− mice, as compared to wild-type, as well as with enhanced differentiation of adult OPCs into differentiated CC1^+^ oligodendrocytes [30]. Interestingly, while NG2^+^ OPC densities were identical in the corpus callosum of wild-type and Cdk2−/− non-injured mice, their reduced proliferation at 7 days post injection (dpi) coincided with a reduction in OPC density in Cdk2−/− mice, as compared to wild-types. In contrast, CC1^+^ oligodendrocyte density increased in Cdk2−/− mice 1 and 2 weeks later, a delay corresponding to the onset of remyelination and the oligodendrocyte/myelin regeneration period in LPC lesions, respectively. However, differences between Cdk2−/− mice and wild-types disappeared by 21 days, when the process of repair was nearly accomplished [30]. Moreover, increased cell cycle exit and oligodendrocyte differentiation in Cdk2−/− mice had a direct impact on the number of remyelinated axons, and on the extent of axonal remyelination, as assessed by g ratio measurements in the lesion (Table 1). The fact that remyelination in wild-type mice levelled with that of Cdk2−/− mice at 21 dpi further underlines that Cdk2 acts as a modulator rather than as an on-off switch of the differentiation/remyelination process. These results are consistent with previous *in vitro* findings showing that Cdk2 loss decreased OPC proliferation [29] and promoted lineage commitment of NG2-expressing progenitors in the adult SVZ, and differentiation of adult neural progenitor cells [39]. In view of these studies, Cdk2 appears as an intrinsic molecular mechanism that controls the timing of oligodendrocyte differentiation *in vivo*. This conclusion is supported by the fact that accelerated differentiation of oligodendroglial cells in the absence of Cdk2 occurred also *in vitro*[30].

Requirement for Cdk2 in adult OPCs could contribute to a better understanding of remyelination failure in multiple sclerosis. In this inflammatory disease, endogenous remyelination exists, but is not efficient in all lesions. Among several possible hypotheses, dysregulation of OPC proliferation has been proposed to play a role in remyelination failure, as demonstrated by reduced OPC proliferation in some lesions, although in others OPCs are numerous, but appear unable to fully differentiate [63]. A previous study also demonstrated that persistent CNS inflammation significantly altered cell kinetics of precursors and stem cells in experimental autoimmune encephalomyelitis, a model of multiple sclerosis [64]. In view of the above mentioned data [30,39], it would be of interest to investigate whether Cdk2 expression is altered in MS and could explain some of the default of remyelination observed in MS lesions.

#### Influence of Cdk4 on PNS myelination and remyelination

Differential requirement of another Cdk, Cdk4, for myelination/remyelination processes, was also described in the PNS. Cell cycle function of Cdk4 is closely related to Cdk2, as both Cdk2 and Cdk4 are activated by their regulatory proteins cyclin E and D, respectively, to sequentially phosphorylate Rb. Although Cdk2−/− or Cdk4−/− mice are viable, Cdk2−/−; Cdk4−/− double KO mice die *in utero* around E15 [65]. Peripheral myelin formation depends on axonal signals that tightly control proliferation and differentiation of the associated Schwann cells [66]. Requirement for three different Cdks was analyzed in Schwann cells using Cdk-deficient mice [67]. Mice lacking Cdk4 showed a drastic decrease in the proliferation rate of Schwann cells at postnatal days 2 and 5. However, proliferation was unaffected at embryonic day 18 which could be due to compensatory mechanisms that are effective during early development. In contrast, ablation of Cdk2 and Cdk6 had no significant influence on postnatal Schwann cell proliferation. Taken together, these findings indicate that postnatal Schwann cell proliferation is uniquely controlled by Cdk4. Despite the lack of the postnatal wave of Schwann cell proliferation, axons were normally myelinated in adult Cdk4-deficient sciatic nerves. Following nerve injury, Schwann cells lacking Cdk4 were unable to re-enter the cell cycle, while Schwann cells deficient in Cdk2 or Cdk6 displayed proliferation rates comparable to controls [67]. No compensatory effects such as elevated Cdk4 levels were observed in uninjured or injured nerves of Cdk2- or Cdk6-deficient mice. So requirement for Cdk4 in Schwann cells, as requirement for Cdk2 in OPCs, seems to be different during developmental myelination compared to pathological event.

#### Differential role of Cdk2 in response to hypoxia

Periventricular white matter injury (PWMI) is a common form of brain injury affecting preterm infants. A major factor that predisposes to PWMI is hypoxia. Influences of hypoxia on oligodendrocytes development were investigated *in vitro* by maintaining purified cultures of OPCs at 21% O_2_ or hypoxia (1% or 4% O_2_) for up to 7 days [68]. 1% O_2_ led to an increase in the proportion of myelin basic protein (MBP)-positive oligodendrocytes after 1 week in culture, and a decrease in the proportion of PDGFRα+ cells suggesting premature oligodendrocyte maturation. Increased expression of the cell cycle regulatory proteins p27^Kip1^ and phospho-Cdk1 was seen as well. Hypoxia appears to interfere with the normal process of oligodendrocyte differentiation by inducing premature OPC maturation that may contribute to hypomyelination in the developing hypoxic brain. The specific role of Cdk2 was not investigated in these studies but giving the role of this kinase in oligodendrocytes, we can suppose that the premature OPC maturation observed in response to increased expression of p27^Kip1^ involves Cdk2 increased inhibition and premature cell cycle exit. The role of Cdk2 in response to hypoxia has been studied in other cell types and it appears that Cdk2 when associated with cyclin A, is involved in hypoxia-induced apoptosis in cardiomyocytes [69]. Indeed, some investigators have interpreted the rapid induction of Cdk2 activity in cardiomyocytes as evidence of post-mitotic cells reentering the cell cycle and undergoing proliferative growth. However, an increasing amount of data indicates that proteins classically thought to be involved in cell cycle regulation also play a critical role in the control of cell death, particularly through apoptotic pathways. Numerous stimuli unrelated to cell cycle progression induce Cdk2 activity and subsequently apoptosis such as irradiation [70], Tumor necrosis factor-α [71], Fas [72], staurosporine [71], hypoxia [69,73], and ischemia [74]. Although Cdk2 activity is elevated during apoptosis, activation of the mitotic processes does not occur even in cells capable of proliferating [72]. Likewise, perturbations of cell cycle progression, without affecting Cdk2 activity, fail to block apoptosis [75] supporting the notion that Cdk2 activation is not simply indicative of aberrant mitosis but, instead, that Cdk2 plays a direct role in cell death.

#### Cell cycle regulatory proteins involvement in oligodendrocyte survival after ischemia

After transient focal cerebral ischemia, cyclin Dl and Cdk4 were selectively expressed in morphologically intact or altered oligodendrocytes and neurons localized to the ischemic tissue. Apoptotic cells were not immunoreactive to these proteins at 46 hours of reperfusion after 2 hours of middle cerebral artery occlusion (I/R injury). However, cyclin D1 and Cdk4 induction is not involved in the onset of astrocyte and microglial cell proliferation in response to ischemia [76]. p21 protein levels in oligodendrocytes and neurons were also found to gradually increase to 250% and 140% of contralateral levels in areas bordering the infarct core up to 6 h following middle cerebral artery occlusion whereas a low level of constitutively p21 was found in nuclei of these cells. In contrast, p53, p27 and cyclin E levels decrease in the infarct core and border areas with time after middle cerebral artery occlusion. The selective up-regulation of p21 in the border zone of a focal ischemic infarct indicates its involvement in an adaptive response to ischemic injury [77]. The potential contribution of Cdk2 to oligodendrocyte survival in response to cerebral ischemia was not investigated in these studies but was observed in other cell type. Indeed, myocardial ischemia activates Cdk2, resulting in the phosphorylation and inactivation of Rb. Blocking Cdk2 activity reduces apoptosis in cultured cardiac myocytes. Genetic or pharmacological inhibition of Cdk2 activity *in vivo* during I/R injury led to a 36% reduction in infarct size, when compared to control mice, associated with a reduction in apoptotic myocytes [78]. These data suggest that Cdk2 signaling pathways are critical regulators of cardiac I/R injury *in vivo* whereas the loss of Cdk2 was shown to have no effect on apoptosis consecutive to focal demyelination in the adult brain [30] confirming the cell specific role of Cdk2.

#### Cell cycle activation following spinal cord injury

Traumatic spinal cord injury (SCI) evokes a complex cascade of events with initial mechanical damage leading to secondary injury processes that contribute to further tissue loss and functional impairment. Growing evidence suggests that the cell cycle is activated following SCI [79-81]. Expression of key cell cycle activator genes, such as cyclin D, Cdk4 and Rb were up-regulated at the early time points (i.e., 4 h and 24 h) after SCI [82]. Upregulation of cell cycle proteins after injury appears to contribute not only to apoptotic cell death of postmitotic cells, including neurons and oligodendrocytes, but also to posttraumatic gliosis and microglial activation. Inhibition of key cell cycle regulatory pathways reduces injury-induced cell death, as well as microglial and astroglial proliferation both *in vitro* and *in vivo.* Treatment with cell cycle pharmacological or endogenous inhibitors like p21 in rodent SCI models prevents neuronal cell death and reduces inflammation, as well as the surrounding glial scar, resulting in markedly reduced lesion volumes and improved motor recovery [82]. In a recent study, Cdk2 and cyclin H protein expression and their association were demonstrated to be increased in proliferating microglia and astrocytes in a rat spinal cord contusion model [83] whereas loss of Cdk2 did not affect proliferation of these cells in response to focal demyelination of the adult brain [30]. Cyclin H regulates cell cycle transitions by forming trimeric Cdk-activating kinase (CAK) complex with Cdk7 and MAT1 that phosphorylates and activates Cdk2. So Cdk2 and cyclin H may play a proliferative role in spinal cord injury.

## Conclusion

In the adult CNS, requirement for Cdk2 appears specific to oligodendrocyte cell type as Cdk2 loss appears to affect only cells giving rise to this lineage. This could explain the differential requirement for Cdk2 in adult germinative regions: although Cdk2 is critical for proliferation and self-renewal of neural progenitor cells in the adult SVZ, it is dispensable for adult hippocampal neurogenesis. Lower Cdk2 expression and loss of compensation mechanisms in the adult could contribute to the progressive decline of proliferation potential and self-renewal of SVZ cells with aging and to their lower regeneration ability. Cdk2 also controls OPC cell cycle progression and is downregulated in adult OPC characterized with low proliferative activity. However, it is not required for normal development of myelinating oligodendrocytes and myelination in the postnatal and adult CNS probably due to compensatory mechanisms only effective during development and early postnatal life. Indeed, loss of Cdk2 has consequences on adult remyelination with decreased OPC proliferation but accelerated differentiation and repair. So, requirement for Cdk2 in OPCs seems to differ during developmental myelination compared to pathological events and this discrepancy could be of relevance for the comprehension of pathological mechanisms and the elaboration of therapeutic strategies specifically targeted to OPCs. Inhibitors of Cdk2 are developed and applied in cancer therapy. Whether such inhibitors would promote remyelination in animal models and whether Cdk2 plays a role in remyelination failure in MS or other myelin disorders remains open questions and deserve further attention.

## Competing interests

The authors declare that they have no competing interests.

## Authors’ contributions

CC drafted the manuscript and designed the figures. AB supervised and corrected the manuscript. Both authors read and approved the final manuscript.
